# Underestimated Factors Regarding the Use of Technology in Daily Practice of Long-Term Care: Qualitative Study Among Health Care Professionals

**DOI:** 10.2196/41032

**Published:** 2023-07-26

**Authors:** Sjors W M Groeneveld, Marjolein E M den Ouden, J E W C van Gemert-Pijnen, Rudolph M Verdaasdonk, Harmieke van Os-Medendorp

**Affiliations:** 1 Research Group Technology, Health & Care, School of Social Work, Saxion University of Applied Sciences Enschede Netherlands; 2 Research Group Smart Health, School of Health, Saxion University of Applied Sciences Deventer/Enschede Netherlands; 3 TechMed Center, Health Technology Implementation, University of Twente Enschede Netherlands; 4 Research Group Care and Technology, Regional Community College of Twente Hengelo Netherlands; 5 Centre for eHealth and Wellbeing Research, Section of Psychology, Health and Technology, University of Twente Enschede Netherlands

**Keywords:** health technology, eHealth, digital health, nurse, nurse assistant, health care professionals, implementation, adoption, acceptance, competencies

## Abstract

**Background:**

Increasing life expectancy is resulting in a growing demand for long-term care; however, there is a shortage of qualified health care professionals (HCPs) to deliver it. If used optimally, technology can provide a solution to this challenge. HCPs play an important role in the use of technology in long-term care. However, technology influences several core aspects of the work that HCPs do, and it is therefore important to have a good understanding of their viewpoint regarding the use of technology in daily practice of long-term care.

**Objective:**

The aim of this study was to identify the factors that HCPs consider as relevant for using technology in daily practice of long-term care.

**Methods:**

In this qualitative study, 11 focus groups were organized with 73 HCPs. The focus group discussions were guided by an innovative game, which was specifically developed for this study. The content of the game was categorized into 4 categories: health care technology and me; health care technology, the patient, and me; health care technology, the organization, and me; and facilitating conditions. The perspectives of HCPs about working with technology were discussed based on this game. The focus groups were recorded and transcribed, followed by an inductive thematic analysis using ATLAS.ti 9x (ATLAS.ti Scientific Software Development GmbH).

**Results:**

Overall, 2 main domain summaries were developed from the data: *technology should improve the quality of care* and *acceptance and use of technology in care*. The first factor indicates the need for tailored and personalized care and balance between human contact and technology. The second factor addresses several aspects regarding working with technology such as trusting technology, learning to work with technology, and collaboration with colleagues.

**Conclusions:**

HCPs are motivated to use technology in daily practice of long-term care when it adds value to the quality of care and there is sufficient trust, expertise, and collaboration with colleagues. Their perspectives need to be considered as they play a crucial part in the successful use of technology, transcending their role as an *actor* in implementation. On the basis of the findings from this study, we recommend *focusing on developing technology for situations where both efficiency and quality of care* can be improved; *redefining the roles of HCPs* and the impact of technology hereon; *involving HCPs in the design process of technology* to enable them to link it to their daily practice; and *creating ambassadors in care teams* who are enthusiastic about working with technology and can support and train their colleagues.

## Introduction

### Background

Health care is facing great challenges globally. On the one hand, there is increased life expectancy [1], resulting in increasing demand for care [2]. In contrast, there is shortage of qualified health care professionals (HCPs) to deliver this care [3]. In the Netherlands alone, it is expected that there will be demand for an additional 137,000 HCPs (mainly nurses and nurse assistants) by 2032 [4]. In addition, there is a shift in the location of care delivery, and people tend to live long in their own homes (ie, aging in place) [5]. This poses challenges for HCPs working in long-term care, who provide care for older people and people with mental or physical disabilities.

Technology is one of the solutions for bridging the gap between the increased demand for care and the number of available HCPs. Examples of such technologies in the long-term care setting are eHealth, robotics, electronic health records, virtual reality, and artificial intelligence. Their use is rapidly expanding, within both cure and care, and it is envisioned that they will influence the autonomy and independence of patients [6]. This increased use of technology has an impact on several aspects of the total health care system. Primarily, it transforms the way in which patients receive and experience care. Furthermore, it alters traditional financing flows, organizational aspects, and even a wide (political) system [7].

Technology also has an impact on HCPs, as they play an important role in its use [7,8]. Several studies focused on the attitude of HCPs regarding a specific type of health care technology [9,10]. Experience [11], enhanced patient care and safety [12-14], and easy-to-use technology that fits within existing workflows [14] lead to a more positive attitude of HCPs regarding technology. Badly designed and nonoptimal functioning of technology [12,13] contributes to negative attitude toward technology.

The focus on the adoption, use intentions, and behavior of a user regarding a specific technology is reflected in scientific studies [15,16]. Although this focus on adoption and acceptance is valuable, it remains as a narrow. The implementation of technology demands a broad look [17,18], as also seen in technology implementation models [7,19], including organizational variables and consequences of use in daily practice.

Particularly within this broad scope, it is important to have a good understanding of the viewpoint of HCPs regarding working with technology, as it potentially influences several core aspects of their work. First, the use of technology can have an impact on workflow [14,20,21] and workload [22-25] and can even contribute to or reduce clinician burnout [26]. In addition, the use of technology can lead to unintended consequences with possible negative outcomes [27]. Examples of unintended consequences include increased complexity, risk of no follow-up of care, and reduction in communication [22]. Furthermore, technology influences the interpersonal relation between HCPs and patients, leading to the loss of personalization [13]; however, it can also serve to improve communication [14,28,29].

### Objective

Thus, although technology can be a solution for bridging the gap between the increased demand for care and the number of available HCPs, it influences several core aspects of the work of HCPs in long-term care. Although some studies focus on patterns of technology adoption by an individual HCP or a specific type of technology, we built upon literature [7,17,19] that emphasizes the need for a more overarching approach to understand how technology influences work in practice. Hence, the research question of this study was the following: which factors do HCPs consider as relevant for using technology in daily practice of long-term care?

## Methods

### Study Design

An inductive qualitative study based on the thematic analysis approach [30] was used because it is the most fitting method as a result of the limited evidence about factors that HCPs consider as relevant for using technology in daily practice of long-term care. Qualitative data were collected using focus groups, a research method suited for discovering perceptions and feelings about a specific topic and discussing them in a team setting [31]. We used a single-category focus group design, adding data until no new insights were gained [31].

### Study Population

This study was conducted on a regional scale in the province of Gelderland in the Netherlands, where 15 long-term care organizations and education institutions worked together in a project focused on improving the digital skills of their employees. HCPs working in these long-term care organizations were invited to participate in this study. The main researcher contacted the care organizations regarding participation. Consequently, the care organizations used convenience sampling to invite participants and to compose a focus group of employees, whereby no detailed demographic data were collected. Some participants already worked together as a team. In general, participants included nurses and nurse assistants, with higher and secondary vocational education, working in a broad range of long-term care settings such as nursing homes for older people and people with mental or physical disabilities. Furthermore, all participants had at least some experience with technology in their work as HCPs.

In this study, 73 HCPs working at 6 different care organizations participated in 11 focus groups. The focus groups consisted of 4 to 11 participants, with most focus groups consisting of 5 to 8 participants, which is seen as the ideal size for this type of study [31].

### Data Collection

The focus group discussions were guided by a nondigital game, which was specifically developed for this study. Each focus group was led by a researcher who acted as a moderator to support the focused discussions. The researcher had no other involvement with the participants. The researcher guided the discussion and was specifically instructed to involve all participants in the discussion, which was also reflected in the design of the game. The focus group discussions had a duration of 60 to 90 minutes and were conducted at the workplace of the HCPs. Participants were able to recontact the researchers through an organization contact person if they wished to share additional views and experiences that had not been shared during the discussion.

### Game Design

#### Overview

**Figure 1:**
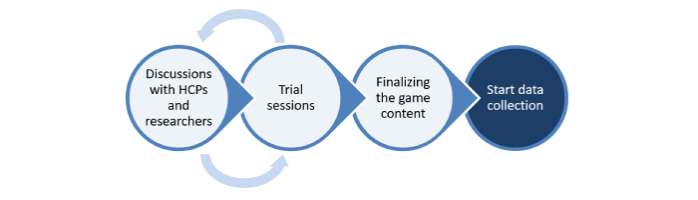
Steps taken in creating the game.

**Figure 2:**
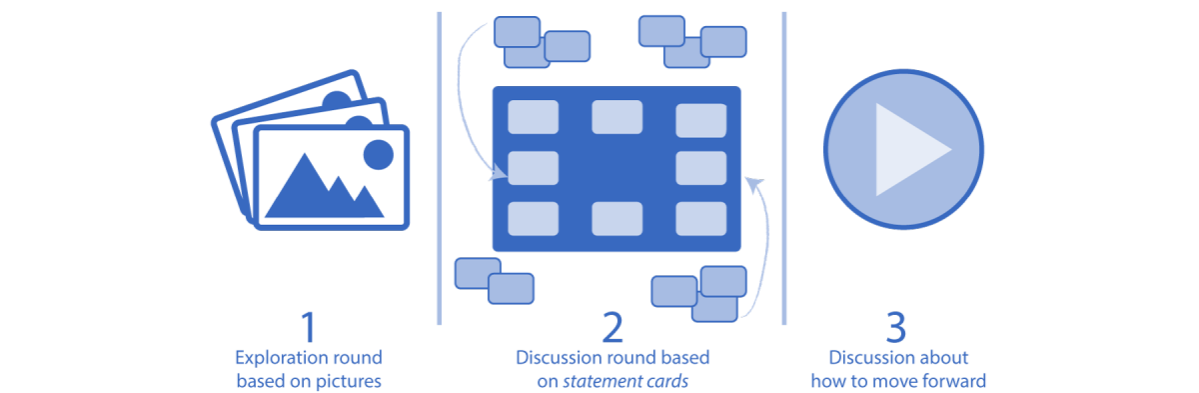
Overview of the three rounds in the game.

The aim of the game was to gain insight into the perspectives of HCPs about working with technology. The content of the game was iteratively formulated in close cooperation with HCPs and involved researchers during several meetings and brainstorms. The game was piloted in multiple trial sessions with both the HCPs and researchers involved, before data collection commenced (Figure 1):

The game consists of 3 rounds: an exploration round based on pictures, a discussion round based on statement cards, and a discussion about how to move forward (Figure 2).

#### Round 1

Participants were asked to choose a photo that reflected their view about health care technology. These photos are a standard collection used to evoke associations on a diverse range of topics. Examples included photos of a field of flowers, 2 hands holding each other, and the cockpit of a plane. Consequently, the researcher would accommodate a discussion about the associations and thoughts that the participants came up with in relation to the photos and their view about health care technology.

#### Round 2

The second round was guided by a set of statement cards (Multimedia Appendix 1). The participants were asked to choose 8 out of 37 cards that they found important in working with technology in long-term care. The content of the cards was divided into four categories: (1) health care technology and me; (2) health care technology, the patient, and me; (3) health care technology, the organization, and me; and (4) facilitating conditions. Every participant received a couple of cards ensuring that everyone was involved in this round of the game. During the process of selecting the cards, the researcher would accommodate a discussion between participants, thereby gaining insights into the motives and thoughts regarding working with technology in long-term care.

#### Round 3

When participants finished their selection of 8 cards, the researcher would ask them what they would like to do or change in their work to accomplish the desired situation. This was done to gain a deep understanding of the opportunities and barriers seen by participants in working with technology in long-term care.

### Data Analysis and Quality Measures

The focus groups were recorded, transcribed, and qualitatively analyzed by 3 researchers using ATLAS.ti 9x (ATLAS.ti Scientific Software Development GmbH). The pictures (round 1) and statement cards (round 2) served as a starting point for the discussions in the focus groups, and the recordings of the discussions were in turn used for qualitative analysis.

**Figure 3:**
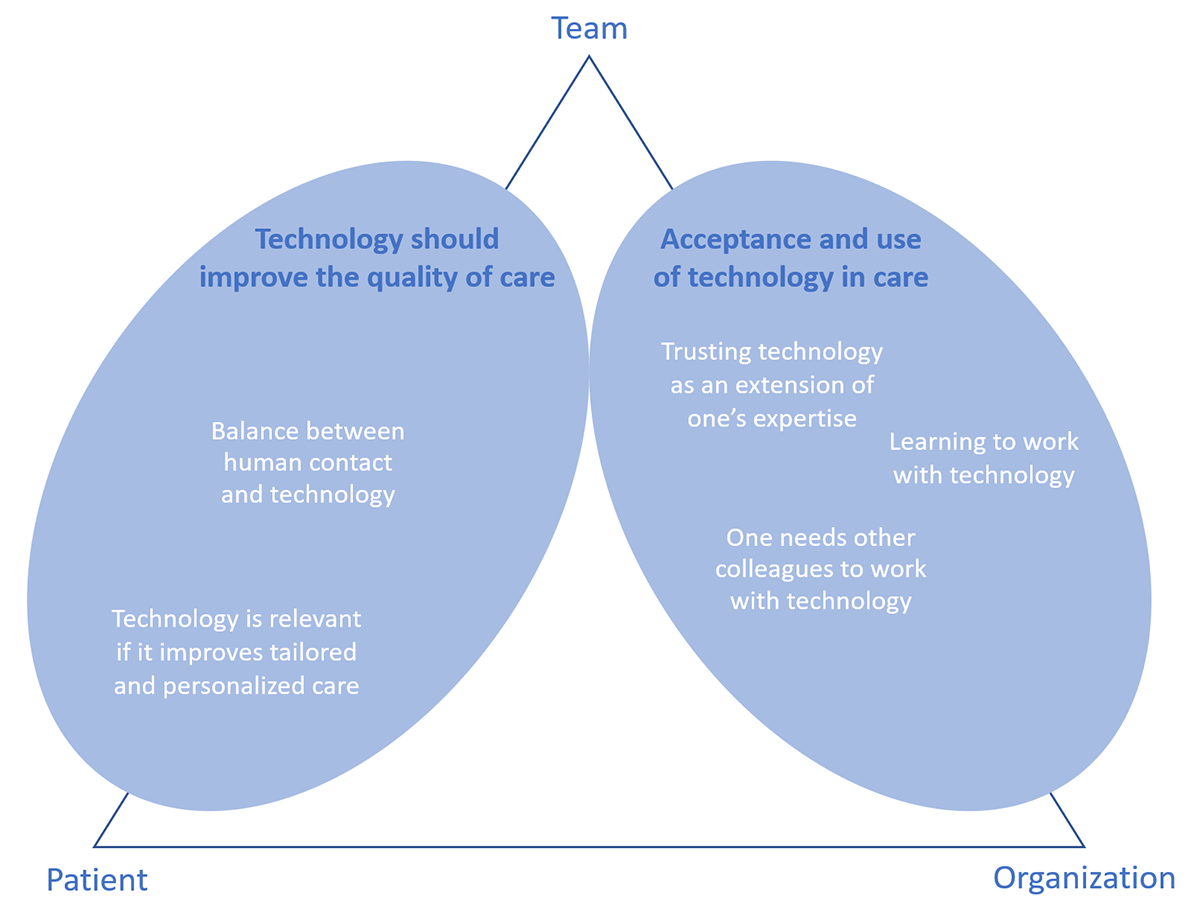
Overview of domain summaries, themes, and layers.

The thematic analysis consisted of 6 phases as described by Braun and Clarke [30]. First, the focus group discussions were transcribed by a research assistant, and transcripts were read and reread to get a first impression of the whole of the session. As a follow-up, initial codes were generated based on the transcript, followed by collating codes into potential themes. These steps were performed by the first author (SWMG), and 2 other researchers checked the process (MEMdO and HvO-M). After all the 11 focus group sessions were coded and potential themes identified (Multimedia Appendix 2), the 3 researchers jointly reviewed the themes, agreed upon relevant overarching domain summaries [32], and generated a thematic map of the analysis (Figure 3). Where necessary, all authors discussed the interpretations, thereby reaching consensus on the definition and naming of the domain summaries and themes. Consequently, a report was produced including, among other things, a selection of quotes that reflected the views of participants on each theme.

During the research process, several quality measures were pursued [33], such as recording the discussions to ensure proper reporting and involvement of multiple researchers in data collection and data analysis. To get a good understanding of the data, all the authors ensured that all insights were covered when discussing interpretations.

### Ethical Considerations, Informed Consent, and Participation

No ethics approval was applied as this study was not subject to the Medical Research Involving Human Subjects Act, participants were not asked to act or to change behaviors, and the questions were not of a drastic nature. The researchers clarified the aim of the study at the start of the focus groups and notified the participants about the audio recording for data analysis. Participants were asked to verbally consent to their participation in this study, were guaranteed anonymity, and could contact the researchers through an organization contact person if they wished to share anything else at a later point. All confidential characteristics, such as names, were anonymized in the transcription process.

## Results

### Overview

On the basis of the thematic analyses, two domain summaries were developed from the data: (1) *technology should improve the quality of care* and (2) *acceptance and use of technology in care*. Both domain summaries consist of several themes; refer to Figure 3 for an overview.

An overarching notion that is recognized throughout the diverse themes was the impression that the use of technology is never an unambiguous task. HCPs see the use of technology as an interplay among patients, teams of HCPs, and the organization of care. These are not themes in themselves but rather aspects that can be seen as layers in gaining a better understanding of the themes. Figure 3 shows a visualization of an overview of domains and themes related to the layers. The domain *technology should improve the quality of care* is about the team of HCPs in relation to the patient, whereas *acceptance and use of technology in care* concerns the team of HCPs in relation to the organization of care. The domains, including themes and examples of quotes, are described in the following sections.

### Technology Should Improve the Quality of Care

#### Overview

During the focus groups, participants emphasized that technology should be used to improve the quality of care. The themes *technology is relevant if it improves tailored and personalized care* and *balance between human contact and technology* were discussed in this regard. If technology did not have an added value for the quality of care or for their clients, HCPs were reluctant to use technology:

I believe that technology shouldn’t replace the professional. It must add something. I am very open to it, and I like technical gadgets, but it should not be at the expense of quality of care.

Focus group 2

#### Technology Is Relevant if It Improves Tailored and Personalized Care

Participants indicated the importance of tailored and personalized care in relation to the use of technology. HCPs stated that the use of a specific technology is only relevant when it fits the needs of a patient:

You have to assess it per client, everyone has a different need for support. We have to discuss if and what technology could work for that specific person.

Focus group 1

Technology should serve the people we work for and not the other way around.

Focus group 4

It doesn’t matter what you or I think about a technology, it is about the patient.

Focus group 5

Furthermore, participants indicated the difference among patients in relation to the use of technology. For instance, age differences or experience of working with technology were seen as important, as this determines what a patient can and is willing to do with technology. There were also some remarks about the safety aspect of the use of technology in health care:

Technology can also offer safety. For instance, we have a motion detector and if someone leaves the department we get a notification.

Focus group 9

Patients are becoming more independent...It gives them a safe feeling they can contact us through a messenger application.

Focus group 8

#### Balance Between Human Contact and Technology

The driving force for the participants to work as HCPs was to help people and to be of significance to them. Personal contact with their patients is an aspect that they love about their job, and technology alters this interpersonal contact. Both drivers of and barriers to this topic were mentioned. On the one hand, technology can be of added value to deliver personalized care and increase quality of care. In contrast, technology could hinder interpersonal contact between professionals and patients, making it more difficult to truly connect with patients:

The problem is that you don’t really want this at all. I chose to work in healthcare, and I want to work with my hands. You don’t want to be busy with these things [technology] at all, you want to work with the people themselves.

Focus group 1

Technology can be of added value for some people, but it also makes people lonelier. If you talk to a video screen, there is no one sitting next to you to drink a cup of coffee with. This feels troubling to me.

Focus group 1

In addition to their own involvement regarding the interpersonal aspects of technology, participants also indicated the influence of substitution of care by technology on the related personal aspects for the clients themselves:

In the future we will have less time on our hands. I do however find it a bit frightening what this will do to human contact. For instance, I don’t see a robot putting an arm around someone in the near future.

Focus group 7

It is a bit troubling but also interesting to think of a robot washing people. I feel it is a bit inhumane.

Focus group 7

### Acceptance and Use of Technology in Care

Participants also emphasized several aspects regarding working with technology in their job. Multiple aspects of *trusting technology* were discussed, and *learning to work with technology* and *collaboration with colleagues* were also topics of discussion.

#### Trusting Technology as an Extension of One’s Expertise

A prerequisite for using technology in HCPs’ work was trust; multiple aspects of this theme were discussed. First, working with new technology requires trust to rely on it. Participants stated that they were insecure about whether the technology would work when needed:

We blindly trust a piece of technology, but we are not sure if it can be trusted.

Focus group 3

Every time I arrive here, I am worried whether or not it will work.

Focus group 4

For me it is also the feeling of being safe, almost everything uses electricity. What if it breaks down, can we still get someone out of bed?

Focus group 7

Another aspect of trust was the lack of confidence in their ability to keep up with the speed of technology innovation. They were afraid of lagging behind if they did not comply with and master these skills. Being a qualified HCP requires new competencies, and not all professionals consciously choose to work with these types of skills:

I know I need to continuously keep up with new developments, but if I don’t succeed I get nervous.

Focus group 3

The last aspect of trust was related to the open character of technology, for instance, in the use of portals for electronic patient records. They were fully aware of this aspect, as family members and informal caregivers can read and follow patient information. If they make a mistake, family members and informal caregivers can see this directly:

You have to think carefully what you report in a client file as family can read along in the new system.

Focus group 1

#### Learning to Work With Technology

If there is enough trust, it is important that HCPs know how to work with technology; participants discussed their preferences about learning how to work with technology. First, they indicated that they would like to know which technologies are available, both within and outside their organization. They need help to structure which technologies are relevant and which can add to the quality of care:

I would find it easy if there was a simple overview to see what is available. In my experience, there is so much information available that I get confused and I am more likely not to use it than I am to use it.

Focus group 10

Second, after HCPs had learned about the available technologies, they indicated the importance of addressing different learning preferences. Some participants want to experiment with technology themselves, whereas some need a colleague to instruct them and others want (written or oral) tutorials:

A manual or instruction video doesn’t work for me, I have to see it, someone has to show it to me.

Focus group 2

I find it important to try it and to see if it works before we buy it and use it.

Focus group 1

Although they found it important, participants emphasized that it takes time to learn to work with technology. In contrast, they expressed that technology can be efficient and saves time if it is used correctly:

It takes time to learn, but in the end it can also save time.

Focus group 2

It is very important that you get time to learn [to work with technology], because in healthcare there is a high workload and then learning gets pushed aside fast.

Focus group 11

I spend a lot of time at the computer for work, which is a waste of time. Me and a colleague spent ten minutes working out how to turn on the screen.

Focus group 4

There were also many remarks regarding the (difference in) age of HCPs and the effect this has on learning to work with technology. For example, the difference between growing up in a digital or nondigital world and thereby gaining competencies to work with technology were discussed. Participants expected that young HCPs would have more skills in working with technology. Finally, some participants said that they were not interested in learning the skills needed for working with technology as they were approaching retirement:

Yes, age is important, it is about what you grew up with. But I also believe it can be learned, no matter how old you are. It is, however, more difficult when you are older.

Focus group 1

I have worked as a nurse for over 40 years. By the time I retire I will have mastered the skills needed.

Focus group 1

Technology can be of added value, but I can’t keep up with it and that’s fine with me at my age.

Focus group 4

#### One Needs Other Colleagues to Work With Technology

Participants indicated multiple aspects of collaboration during the use of technology in their work. Colleagues were seen both as an important source of information and as a source of support when needed:

I don’t know everything, but I know I can always ask a colleague. Together we will find a way. I find it very important that we are there to support each other.

Focus group 3

Participants indicated the need for a team member who is able to support them with the use of technology, a so-called expert or ambassador. This person should not replace a technical or innovation department, but they could transfer knowledge from these departments in a more accessible way to the care team:

I prefer to have a colleague sitting next to me and explaining what I should do in a way that I can understand.

Focus group 11

I would like to have someone in my team that I can consult in case I have any questions. I prefer to ask questions instead of searching on the internet or folders.

Focus group 9

The information and communications technology (ICT) department colleagues were specifically mentioned. Participants perceived their help as supportive as they were able to solve problems quickly and effectively. However, HCPs also stated that some ICT professionals do not understand the context of health care and do not acknowledge the fact that not all HCPs are technical and thereby fail to give personal support to them adequately:

People working at the ICT department are technical people, they think I am stupid.

Focus group 6

If I call the ICT department they take over my pc and fix my problem. However, this is way too fast for me to understand.

Focus group 11

Finally, participants commented about the collaboration between colleagues at the board and strategic level and HCPs. They indicated the importance of fine-tuning between goals and plans made at the organizational level and the use of technology in practice:

It is about the way they make the plans; we need to participate. Now they develop plans from behind a desk without knowing how it works in practice.

Focus group 9

The colleagues who make the decisions are not aware of our situation. They should come and talk to us.

Focus group 11

For us it is not clear what the policy of our organization is regarding the use of technology, in terms of communication it could be a lot better.

Focus group 9

## Discussion

### Principal Findings

Our findings showed that HCPs are willing to use technology if it improves tailored and personalized care and when it is an extension of their expertise. Furthermore, the balance between human contact and the use of technology is of utmost importance to them. We also found that sufficient trust, expertise, and collaboration with colleagues in using technology in daily practice are important aspects of working with technology from an HCP perspective. A fit between technology, patient, team of HCPs, and the organization of care is important. This means that technology is context dependent and a one-size-fits-all approach is not successful.

Although a vast amount of research is being conducted focused on the adoption, use intentions, and behavior of a user regarding a specific technology [15,16], this study builds upon previous studies of others who took a broad system perspective about technology in health care consistent with technology implementation models [17,19]. This study adds the perspective of experienced HCPs about working with technology in long-term care to this broad scope. In this way, this study provides a detailed insight into the thoughts and motives of HCPs regarding working with technology in long-term care, transcending their role as an actor in using technology.

HCPs find it important that technology adds to the quality of care, an aspect that is also seen in several other studies where it sometimes was defined as the *enhancement of patient care and safety* [12-14]. In this study, HCPs indicated that technology should improve tailored and personalized care. Some overlap is recognized with the *performance expectancy* aspect in technology acceptance models, where it is originally defined as “the degree to which an individual believes that using the system will help him or her to attain gains in job performance” [15]. Improving tailored and personalized care, as indicated by HCPs in this study, could be seen as a form of gain in job performance; however, it is more focused on output quality than on effectiveness or productivity. This overlap was also found by Holtz and Krein [34], who explained productivity in terms of a high standard of care. Moore et al [35] found that using technology actually influenced the distribution of available nursing time. From a management perspective, it is conceivable that technology can be introduced to achieve great work efficiency, but as seen in the results of this study, it can also lead to an aversion to working with technology among HCPs, as technology, in this sense, does not necessarily add to the improvement of tailored and personalized care.

In relation to the quality of care, HCPs also indicate that they are searching for a good balance between human contact and the use of technology, referred to as balancing the human element with technology [36]. A possible explanation for this might be that HCPs find interactions with other people to be important [37], and using technology could lead to less contact, resulting in a negative attitude toward technology [9,13]. A recent systematic review of patients and HCPs’ perspectives toward technology-assisted diabetes self-management education also concluded that technology should not replace or hinder human contact [38], with some studies also identifying situations where technology could benefit the process of communication [28,29].

Both these findings raise questions regarding the use of technology in relation to efficiency and quality of care. There is a shortage of qualified HCPs, and the use of technology could possibly help to overcome this challenge by working more efficiently. However, if HCPs maintain the current level of human contact and only use technology when it adds to the quality of care, it is questionable whether technology will actually form a solution. This means that the role of HCPs will probably change.

Consistent with previous studies [7,15,16], several aspects regarding working with technology (such as the role of the organization, influence of the involved patient, knowledge and skills needed to use technology, age, and social influence) were recognized. In this study, these aspects were discussed from the perspective of HCPs. We found that HCPs prefer a direct colleague who can support them with the use of technology (an ambassador). This is consistent with the work of Cain and Mittman [39], who identified the importance of opinion leaders in the diffusion of innovations within health care and others who acknowledge the benefits of clinical champions [40,41]. The preference for an ambassador can also be seen as a form of *social influence* [15]*.* This study adds indications to use this aspect proactively to stimulate the use of technology by HCPs by creating an ambassador in the team who is enthusiastic about working with technology. There was a strong preference for an ambassador with the same background as the HCP. A possible explanation for this might be found in the in-group and out-group theory [42], as social interactions within the in-group are “more predictable and understood.” In practice, ICT colleagues are available to support with technical issues. However, HCPs indicate that these colleagues are not able to support adequately because they do not understand the context and situation of an HCP.

Although this study indicates several aspects regarding working with technology, these can (and probably will) change over time. The workforce is continuously evolving, and new generations are finding their way into health care. New HCPs, for instance, those belonging to generation Y (born between 1982 and 2005), are seen as more experienced in working with technology and are considered as having more skills to do so than earlier generations [43]. Furthermore, at this point, there is growing attention on the use of technology in the educational programs of HCPs [44]. In this way, some of the found issues may become less relevant in the course of time as increasing number of professionals become used to working with technology. However, it is important to take the distinction between the use of technology in personal life and in a professional way into account. Furthermore, not only is the workforce changing but technology, organizations, and society as a whole are also continuously evolving and transforming health care. Therefore, we also recommend providing support and training to the current workforce, especially because lifelong learning is considered to be important for practicing nurses [45] and the lack of knowledge and skills is seen as one of the barriers to implementation [25]. A recent scoping review indicated several subjects that should be part of nursing training to enable HCPs to effectively use technology [46] and integrate it into care delivery.

### Strengths and Limitations

A strength of this study is the broad involvement of HCPs. The varied participants included nurses and nurse assistants on both higher and secondary vocational education levels. As the care organizations sampled their employees, no detailed demographic and other work-related data were collected on an individual level, and there is a lack of data about nonparticipation. Therefore, it is important to bear in mind that there is a possible selection bias, which could have influenced the results. However, this approach made it possible to get a complete and diverse idea about which factors are relevant to HCPs working in long-term care in a similar composition to their regular teams. The COVID-19 pandemic has accelerated the use of technology in health care [47]; some of our results may have changed owing to this fact, as data were collected before the pandemic (January 2019 to September 2019).

From a methodological perspective, it is worth noting that this study demonstrates the possibility of using a game as a research instrument to discuss thoughts and motives in a focus group setting. The game is a useful tool to stimulate discussion because it invited the participants to share their views in an accessible and playful manner. For further development of the game as a tool, it is recommended that attention should be given to the design in relation to possible information bias.

### Implications for Practice

An essential follow-up to this study is the translation of the results into practice. On the basis of the findings of this study, it is recommended that the following aspects should be considered*—the development of technology for situations where both efficiency and quality of care can be improved*. An example of such technology is smart incontinence material [48], where a sensor feels whether a person needs to be changed. This prevents HCPs from having to regularly check the incontinence material, resulting in both efficiency (time saving) and quality of care (avoiding the need for unpleasant checks). Furthermore, it is recommended to *redefine the roles of HCPs* and the impact of technology on their role [49]. Working with technology implicates a renewal of tasks between humans and technology and thereby alters the traditional role of the HCP (ie, balance between human contact and technology). In addition, we recommend *involving HCPs in the design process of technology* to enable them to provide input into the design process and link it to their practice. HCPs can provide suggestions about how to improve technology features [24]. Technical guidelines should be followed, and the practical components of using that technology by HCPs, such as integrating into care pathways, should be incorporated to maximize the chance of successful implementation of technology [7].

Finally, we recommend *creating ambassadors in care teams* who are enthusiastic about working with technology, *supporting and training* the current workforce in working with technology, and incorporating it into educational programs for future HCPs.

### Future Studies

The results obtained from this study should be further examined in the future using a quantitative design and a large sample of HCPs, thereby evaluating their completeness. It would be valuable to describe several case studies of care organizations that implement the suggestions and thereby develop scenarios for implementation. Every context is different, and by describing these case studies, more insight can be gathered about the influence of this aspect. Furthermore, technology, organizations, and society are continuously changing, and future studies should take this into account as this can influence the perspective of HCPs.

### Conclusions

This paper presents underestimated factors regarding the use of technology in daily practice of long-term care from the perspectives of HCPs. HCPs want to use technology in long-term care when it adds value to the quality of care and there is sufficient trust, expertise, and collaboration with colleagues on using it in daily practice. The outcomes of this study clearly advocate taking the perspectives of HCPs into account as they are a crucial part in the successful use of technology, transcending their role as an *actor* in implementation.

## Appendix

Multimedia Appendix 1 Statement cards used in the focus group.

Multimedia Appendix 2 Code tree.

## Abbreviations

HCP: health care professional
ICT: information and communications technology
