# Supplementary material for: Underestimated Factors Regarding the Use of Technology in Daily Practice of Long-Term Care: Qualitative Study Among Health Care Professionals
Source: JMIR Nurs. 2023 Jul 26;6:e41032. doi: 10.2196/41032 (PMC10413233; doi:10.2196/41032)
Supplement: Multimedia Appendix 1 [file nursing_v6i1e41032_app1.pdf]

Appendix 1: Statement cards used in the focus group

Healthcare technology and me	
I use social and other media to discover or share experiences of using of healthcare technology.	I set a good example to others with my approach to using healthcare technology.
I consciously assess how well a certain aspect of healthcare technology has worked for me.	I am aware of the possibilities, strong and weak points of the healthcare technologies which I use.
I experiment with new healthcare technology.	I have every confidence in the healthcare technology which I use.
I am familiar with healthcare technology and I can explain its added value.	I have attended a conference on healthcare technology.
I follow developments in the field of healthcare technology.	
**Healthcare technology, the patient and me**	
I work in collaboration with healthcare users to improve healthcare technology.	If a health care user is using a form of healthcare technology I make sure that I understand how it works and how I can use it properly.
I can refer a care user to reputable websites which offer information on their illness, condition or treatment.	I can explain to a care user how a particular healthcare technology will benefit them.
I look for and use the available healthcare technology to meet the individual needs of those requiring health care.	I estimate for the individual care user whether communication through healthcare technology is suitable for them.
I can use data obtained from measuring equipment or digital health records for treatment of a care user.	I encourage care users and informal carers to use healthcare technology.
**Healthcare technology, the organization and me**	
In my team, we encourage each other to use healthcare technology as part of the care we provide..	I work in collaboration with colleagues to improve healthcare technology.
I take part in online discussions to share experiences with other healthcare professionals.	I use my knowledge of healthcare technology to help my colleagues.
I try to find appropriate practical examples of healthcare technology when my own team cannot find a solution to a problem.	I know someone in my team who is good at using healthcare technology and who can answer my questions.
I am aware of the security measures taken by my organization relating to healthcare technology and I stick to these rules.	I use healthcare technology to improve the efficiency of my organization.
**Facilitating conditions**	
During training courses and training programs, we regularly discuss how healthcare technology can be used to improve care for patients.	My supervisor regularly discusses with me how to use healthcare technology and implement it in our care package.
My organization has a clear policy on healthcare technology and I know what we want to achieve in this area.	The use of healthcare technology is regularly discussed during our team meetings.
I know who I can ask for advice if our team wants to introduce new healthcare technology for care users or for ourselves.	My supervisor leads by example in using healthcare technology.
I am given enough time to learn how to use new healthcare technology, my questions are answered and my complaints or suggestions are taken seriously.	My organization provides enough money and time for training its staff to keep up-to-date with advancements in healthcare technology.
My organization provides enough instructions, training videos and manuals that are easy to understand and help me to use healthcare technology.	If healthcare technology does not work the problem is solved quickly.
I feel that I am given enough room to experiment with healthcare technology.	There are colleagues available for technical support who can answer my questions and provide me with help.
