# Supplementary material for: Underestimated Factors Regarding the Use of Technology in Daily Practice of Long-Term Care: Qualitative Study Among Health Care Professionals
Source: JMIR Nurs. 2023 Jul 26;6:e41032. doi: 10.2196/41032 (PMC10413233; doi:10.2196/41032)
Supplement: Multimedia Appendix 2 [file nursing_v6i1e41032_app2.pdf]

Appendix 2: Code tree

Domain	Theme	Code
Technology should improve the quality of care	Technology is relevant when it improves tailored and personalized care	Client ambassador<br>Client is evolving<br>Dependable on: client<br>Dependable on: age of client<br>Dependable on: situation<br>Take efficiency into account<br>Take safety into account<br>Understanding technology to help client
	Balance between human contact and technology	Take added value of technology into account<br>Personal contact<br>Substitution of care<br>Depends on previous experiences<br>Use of technology takes time
Acceptance and use of technology in care	Trusting technology as an extension of your expertise	Afraid technology won't work<br>Afraid of consequences when breaking rules<br>Relatives can read along<br>Dependable on: Trust
	Learning to work with technology	Anxiety for new things<br>Influenced by: Age of HCP<br>Influenced by: High workload<br>Influenced by: Retirement in sight<br>Influenced by: Speed of change<br>Influenced by: Use of technology takes time<br>Instruction<br>It takes time to learn to work with technology<br>Dependable on: Knowledge of technology<br>Dependable on: Learning preferences<br>Need to keep up<br>Not understanding technology<br>Trying technology out
	You need other colleagues to work with technology	Colleagues help each other<br>Complaints<br>Coordinating: colleagues<br>Coordinating: organizations and practice<br>Coordinating: other disciplines<br>Coordinating: social media<br>Ambassador (technology) in team<br>ICT: contact<br>ICT: reachability<br>Stimulate each other in team<br>Who decides which technology is used
